# AnABlast: a new *in silico* strategy for the genome-wide search of novel genes and fossil regions

**DOI:** 10.1093/dnares/dsv025

**Published:** 2015-10-21

**Authors:** Juan Jimenez, Caia D. S. Duncan, María Gallardo, Juan Mata, Antonio J. Perez-Pulido

**Affiliations:** 1 Centro Andaluz de Biología del Desarrollo, Universidad Pablo de Olavide de Sevilla/CSIC, Sevilla, Spain; 2 Department of Biochemistry, University of Cambridge, Cambridge, UK

**Keywords:** *Schizosaccharomyces pombe*, new genes, *in silico* annotation tool, genome evolution, fossil DNA sequences

## Abstract

Genome annotation, assisted by computer programs, is one of the great advances in modern biology. Nevertheless, the *in silico* identification of small and complex coding sequences is still challenging. We observed that amino acid sequences inferred from coding—but rarely from non-coding—DNA sequences accumulated alignments in low-stringency BLAST searches, suggesting that this alignments accumulation could be used to highlight coding regions in sequenced DNA. To investigate this possibility, we developed a computer program (AnABlast) that generates profiles of accumulated alignments in query amino acid sequences using a low-stringency BLAST strategy. To validate this approach, all six-frame translations of DNA sequences between every two annotated exons of the fission yeast genome were analysed with AnABlast. AnABlast-generated profiles identified three new copies of known genes, and four new genes supported by experimental evidence. New pseudogenes, ancestral carboxyl- and amino-terminal subtractions, complex gene rearrangements, and ancient fragments of mitDNA and of bacterial origin, were also inferred. Thus, this novel *in silico* approach provides a powerful tool to uncover new genes, as well as fossil-coding sequences, thus providing insight into the evolutionary history of annotated genomes.

## 1. Introduction

The development of new methods for identifying protein-coding genes and pseudogenes in genome sequences is one of the major challenges in the genomic age. In addition to the extraordinary support given by experimental approaches, computational analysis is an essential task for protein-coding gene identification in present genome biology.^1,2^ Current gene prediction algorithms perform a number of limited but standard steps, including identification of open reading frames (ORFs) and predicted sequence analysis to assign putative functions by similarity to known proteins, motifs, and/or structures in databases.^3–9^ However, even though gene prediction and annotation software are increasingly efficient, small protein-coding genes and complex intron-containing genes may still escape detection by standard *in silico* analysis.

Recent experimental data from ‘omic-technologies’ generated in model organisms are creating the possibility to accurately update published genome annotations.^10^ The fission yeast *Schizosaccharomyces pombe* is one of these model organisms.^11^ The 12.5 Mb *S. pombe* genome was among the first genomes that were entirely sequenced. Based on standard computational prediction, together with experimental data, 4,824 genes and 31 pseudogenes were initially annotated.^12^ However, while >95% of the genome is transcribed,^13–15^ only 55% of the genome corresponds to annotated coding sequences,^12,16^ raising the possibility that more protein-coding genes await to be discovered. Accordingly, high-throughput cDNA sequencing (RNA-Seq) and mass spectrometry-based proteomic approaches^14,17–19^ increased this initial number to the 5,123 protein-coding genes that are currently annotated in PomBase (http://www.pombase.org), the *S. pombe* database.^20^ Some pseudogenes were also reassigned as genes, leaving 29 pseudogenes annotated at present. More recently, the use of ribosome profiling (Ribo-Seq) to investigate the translational landscape of meiosis added further 46 new protein-coding genes to the fission yeast genome.^18^ Most of these new findings correspond to genes coding for small polypeptides and/or coding sequences lacking significant alignments in databases, highlighting that the *in silico* identification of this set of coding sequences is still challenging in genome annotation.

The identification of similar proteins through BLAST analysis is one of the most useful strategies in annotation tasks. Finding significant alignments facilitates assignment of putative functions to query amino acid sequences through the identification of related proteins in databases. Interestingly, non-significant alignments (below the significant threshold) are often found in conventional BLAST searches. In polypeptides derived from the electronic translation of non-coding DNA, such alignments occur by chance. In polypeptides from coding DNA, however, in addition to random matches, non-significant alignments may also underlie small amino acid patterns representing random footprints of common ancestors.^21,22^ Since protein-coding genes mostly arise from previous ones during evolution, even highly divergent coding sequences may harbour ancient footprints of common ancestral proteins to be found among the millions of proteins available in databases. These footprints are not present in non-coding DNA sequences.^22^ Thus, alignments accumulated in predicted amino acid sequences might provide a method to discern coding from non-coding DNA, a new strategy that may overcome limitations of current *in silico* algorithms.

To use this strategy for the identification of coding regions at genome level, we developed a computer program, named AnABlast, which generates profiles of accumulated alignments in query amino acid sequences using a low-stringency BLAST strategy. To test its potential use as a gene-finder algorithm, a genome-wide search of coding sequences was carried out in fission yeast, and Ribo-Seq data used to validate protein-coding regions were highlighted by AnABlast profiles.^18^ Overall, several new genes and ancient coding sequences were efficiently identified by using AnABlast in the extremely well annotated *S. pombe* genome. The strategy was also successfully applied in a genomic region of the nematode *Caenorhabditis elegans*, suggesting that this novel tool may be applicable to the *in silico* analysis of any other sequenced genome.

## 2. Materials and methods

### 2.1. Search strategy

We have designed a computational workflow, here named AnABlast (**An**cestral-patterns search through **A BLAST**-based strategy), to search for putative coding sequences in genomic regions. Briefly, to search for new coding regions in a genome, all inter-exon DNA sequences of the genome (intergenic and intronic regions) were translated in all six frames, masking stop codons with ‘X’ characters; in this way, the analysis become independent of traditional ORF signals. The algorithm then executes a BLASTP search with the six resultant amino acid sequences (as BLASTX, although it can be parallelized into six threads to accelerate the process) and the UniRef50 database (2014_02 version). The UniProt Reference Clusters (UniRef) provide clustered sets of sequences from virtually all the published protein sequences,^23^ being the clusters from UniRef50 built with amino acid sequences having at least 50% sequence identity. AnABlast only uses the representative member from each UniRef50 cluster, and thus, it only considers non-similar sequences at 50% identity threshold (avoiding biases coming from sequence redundancy).

**Figure 1.**
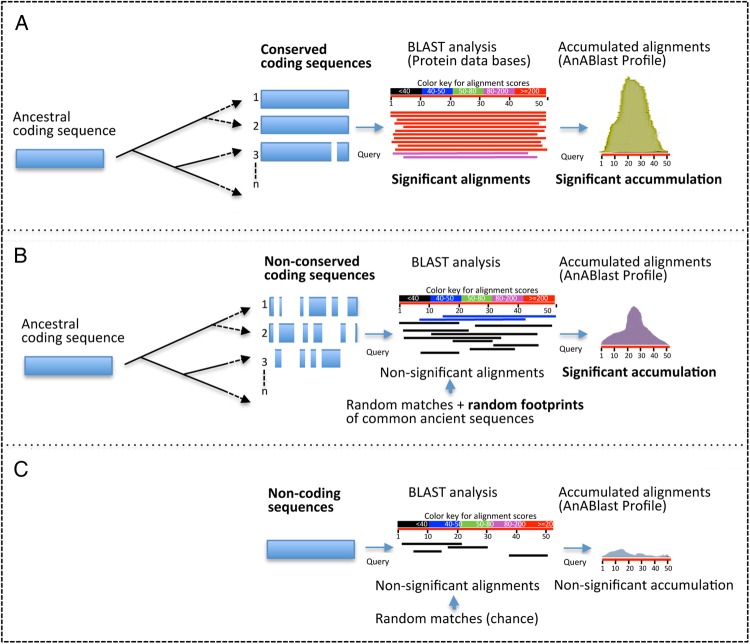
Schematic representation of the AnABlast strategy for the identification of coding sequences. (A) In conserved proteins, conventional BLAST analysis of query sequences usually generates a number of significant alignments that allow the identification of coding sequences. The accumulation of these alignments along the query sequence (AnABlast profile) generates prominent peaks that also allow the easy identification of conserved coding regions. (B) In non-conserved sequences, BLAST search generates non-significant alignments, but AnABlast profiles highlight coding regions by the significant accumulation of these alignments, generated by random matches as well as from random footprints of common ancestors. (C) Non-coding sequences lack common ancestors in protein databases, and non-significant alignments may only occur by chance. In this case, alignments rarely accumulate in particular regions. This figure is available in black and white in print and in colour at *DNA Research* online.

BLAST analysis efficiently identifies homologous sequences through highly significant alignments.^3^ However, because a high frequency of low significant alignments may reveal stretches of ancestral regions,^21,22^ we used a low-stringency BLAST strategy to include alignments beyond a significant similarity, a key step in our strategy to identify putative coding sequences with a biological and/or evolutionary significance (Fig. 1). To this end, AnABlast uses non-restrictive parameters including a low bit score threshold for considering hits. Bit score represents a normalized value that allows comparison of results from different assays. Because bit score thresholds are not allowed in BLAST tools, low bit score values (from 25) were established by using high *E*-values (up to 2,00,000). Using the appropriate scoring matrix during the BLAST search (among available BLOSUM and PAM standard matrices) can improve AnABlast sensitivity. In this study, the BLOSUM90 matrix was selected to favour the search of high identity sequences in very short alignments, enough to be considered protomotifs^21^ or ancient footprints of protein sequences.^22^ The low-complexity filter SEG is also used to avoid sequence repeats, thereby improving specificity. Sequence alignments were trained with coding sequences from the analysed genome (data not shown), and a bit score threshold (given by the score matrix and the search space size) that better highlighted coding regions was used. In the *S. pombe* genome, a threshold of 30 in the score alignment was optimal in our search strategy, corresponding to an *E*-value of ∼12,000 (the default *E*-value for searching significant hits is usually 10). Finally, up to a maximum of 10,000 alignments by query is allowed.

**Figure 2.**
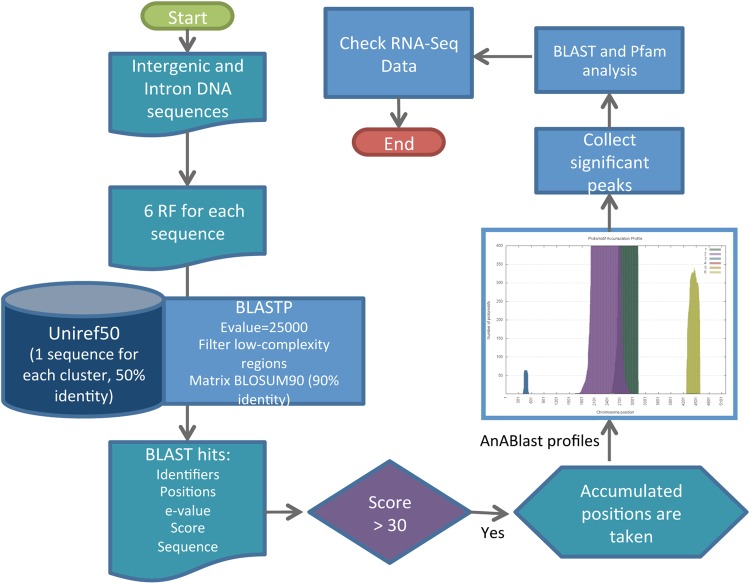
AnABlast algorithm (schematized flux diagram) for coding sequence identification in a genome-wide search strategy. Amino acid sequences obtained from the six reading frames (RF) of each DNA inter-exon region are subjected to BLAST search against a protein database that minimizes redundancies (uniref50). After low-stringency alignment parameters (optimal bit score threshold: 30), hits are taken at each position of the query amino acid sequence, generating a profile of accumulated alignments along the sequence (AnABlast profile). Significant peaks in the generated profiles (higher than 70 in our *S. pombe* genome analysis) were selected, and the corresponding amino acid sequence further analysed by conventional BLAST and Pfam search. Ribo-Seq data in the identified genomic region of AnABlast peaks are also analysed. This figure is available in black and white in print and in colour at *DNA Research* online.

In a second step, AnABlast takes the positions from the acquired hits and counts the number of alignments (belonging to different clusters) that matches each position. As a result of this analysis, AnABlast yields a profile where discrete peaks highlight specific regions in the genome. Independently of the level of significance of each particular alignment, the high number of sequence alignments accumulated at a specific strand and reading frame reveals a significant region in terms of biological function and/or evolution.^21,22^ Under the above parameters, randomized and non-coding sequences yielded AnABlast profiles with peaks rarely reaching a maximum of 70 accumulated sequences. In this study, a cut-off threshold of 70 accumulated sequences was applied to optimize the identification of putative present and fossil-coding regions. The AnABlast algorithm is summarized in Fig. 2 and can be tested by downloading its different modules from http://www.bioinfocabd.upo.es/ab.

### 2.2. Analysis of the selected sequences

For all analysis, *S. pombe* annotations, protein and DNA sequences, and RNA expression data available from PomBase (http://www.pombase.org/) and *C. elegans* (https://www.wormbase.org/) on 1 February 2014 were used. The amino acid sequence delimited by each AnABlast peak was further studied manually by using BLAST^3^ and Pfam^5^ from their web applications (https://blast.ncbi.nlm.nih.gov/ and http://pfam.xfam.org/, respectively). To determine expression levels in AnABlast domains, genome-wide transcriptome Ribo-Seq data from proliferating and sporulating cells.^18^ Ribo-Seq and AnABlast data were visualized with the Integrated Genome Viewer.^24^ Genome-wide RNA-Seq data from cells subjected to temperature or nutritional stress^14,19^ were also used (available at http://www.pombase.org/).

## 3. Results and discussion

### 3.1. Setting up optimal AnABlast parameters

**Figure 3.**
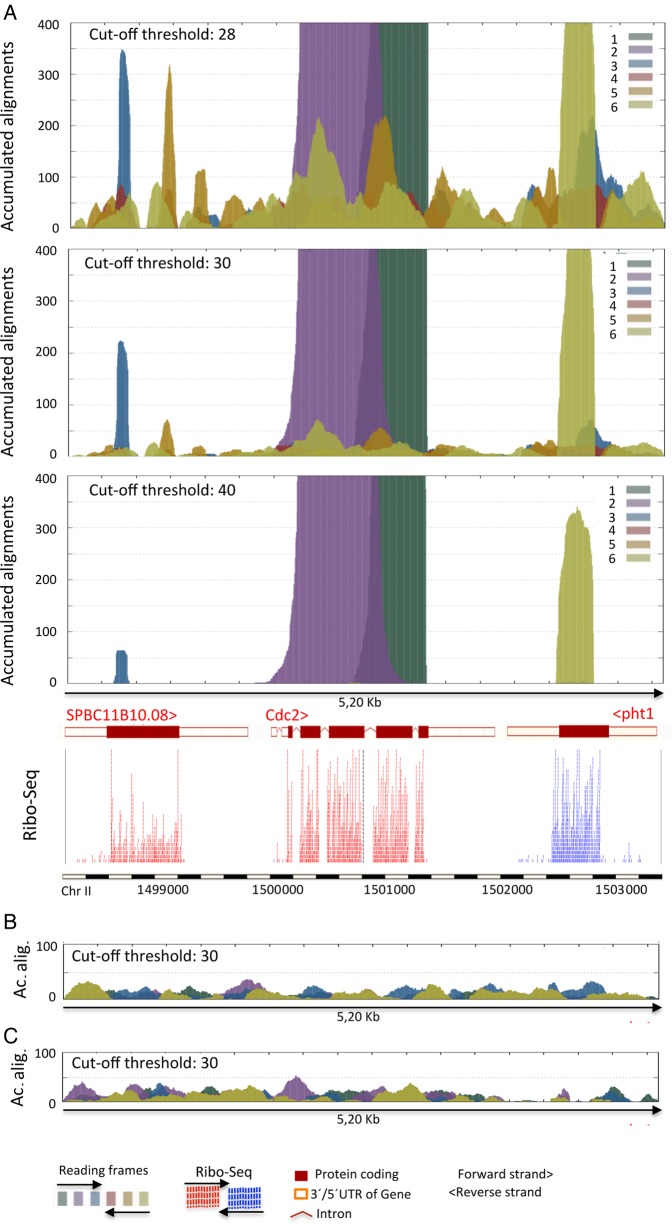
AnABlast profiles (accumulated alignments) in the Chr II: 1498400-1503600 genomic region containing the well-characterized *cdc2* coding sequence and its flanking SPBC11B10.08 and *pht1* genes (Pombase annotations). (A) Accumulation of alignments obtained in different score cut-off threshold from BLAST results (indicated) allows the establishment of optimal parameters for genome-wide search of coding sequences with AnABlast algorithms. Ribo-Seq data are used to confirm accuracy of AnABlast predictions from protein sequences encoded in the three possible reading frames in the forward (colour codes 1–3) and the reverse (colour codes 4–6) strand. (B) Representative AnABlast profile (score threshold: 30) obtained from a randomized DNA sequence of this genomic interval. (C) AnABlast profile (score threshold: 30) from the reverse DNA sequence (lacking biological significance in terms of protein coding) of this genomic interval. This figure is available in black and white in print and in colour at *DNA Research* online.

The significant accumulation of amino acid sequence alignments produced by AnABlast analysis may be useful to identify DNA-coding sequences of biological and/or evolutionary significance (Figs 1 and 2). To establish optimal AnABlast parameters for the accurate identification of coding sequences, we first analysed a genomic DNA region encoding known proteins. The *S. pombe* genome interval containing the universal protein kinase Cdc2 (encoded in the forward strand) and its flanking genes SPBC11B10.08 (forward strand), and *pht1* (reverse strand) was used to this purpose. All six-frame translations of this DNA interval were obtained. To allow the complete analysis of the corresponding amino acid sequence independently of existing ORFs, stop codons were ignored (masked with ‘X’). As expected, AnABlast analysis produced a profile of discrete peaks that matched regions of annotated exons and RNA expression patterns (Fig. 3). Setting optimal thresholds in AnABlast parameters (bit score threshold in sequence alignments and cut-off threshold of accumulated sequences) is nonetheless a key question. The use of very low-stringency parameters to generate BLAST alignments (score = 28) would favour the identification of highly divergent coding sequences in our AnABlast strategy. However, the background noise caused by random matches at this permissive condition makes it difficult to discard false positives obtained by chance in non-coding sequences (Fig. 3A). On the other hand, setting of high alignment cut-off thresholds (score = 40) resulted in the efficient highlighting of only those coding regions with a significant similarity to sequences in databases, a pool of genes that are easily identified by conventional methods. By using a threshold of 30 in the score alignment, AnABlast peaks (accumulated sequences) generated in non-coding regions rarely reached the value of 70, while coding regions accumulated hundreds of sequences, yielding easily identifiable peaks (Fig. 3). Thus, a score alignment threshold of 30 in the BLAST search and a cut-off threshold of 70 in alignments accumulation were used as standard parameters for the selection of significant peaks identifying putative coding sequences.

Virtual translation of non-coding DNA generates random protein sequences. To better estimate false-positive rates under these parameters, we randomly shuffled the DNA sequence of the analysed genome interval. No significant peaks were found in 50 randomized sequences tested (see Fig. 3B), suggesting that significant peaks rarely occur by chance with the selected settings. Similarly, no significant peaks were observed when analysing the reverse 3′-5′ sequence (Fig. 3C).

Classic frameshift alignment analysis aids in the identification of frameshifts in DNA sequences.^25^ As AnABlast profiles identify coding sequences in the different reading frames, coding frameshifts can be also easily inferred from this computational analysis (see Cdc2 exons in Fig. 3A).

### 3.2. AnABlast analysis of inter-exon regions in the entire *S. pombe* genome

To perform a genome-wide search of new coding regions in fission yeast, we analysed all nucleotide sequences between every two adjacent annotated exons, accounting for a total of 5,870 intergenic regions and 4,782 introns. AnABlast profiles were generated for each of the six-frame translation sequences of these inter-exon regions. Among them, 158 regions contained at least one significant peak and were selected for further analysis. As a negative control, we randomized the corresponding DNA sequence in each of the analysed inter-exon regions. With this data set, only 19 significant peaks were obtained (AnABlast profiles obtained both in intergenic sequences, and its randomized versions are available at http://www.bioinfocabd.upo.es/anablast/supplementary_data.html). Thus, on average, >85% of the selected AnABlast peaks are expected to identify true-positive coding sequences.

**Figure 4.**
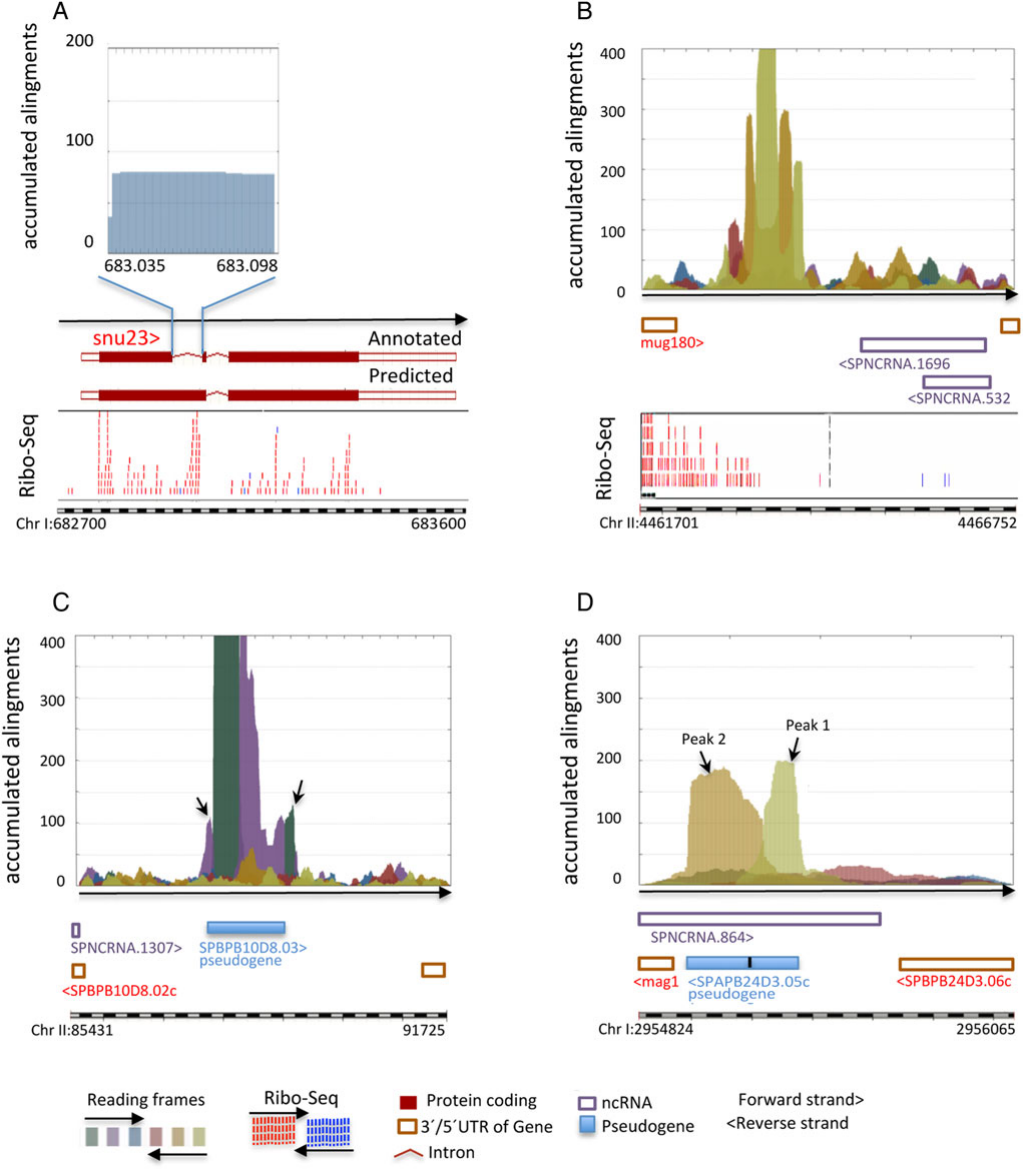
AnABlast profiles suggesting modifications in annotated intron and pseudogenes. (A) Profile generated in the first intron (Chr I: 683035-683098, forward strand) of the *snu23* gene. Ribo-Seq data of the *snu23* gene region are shown. (B) Profile identifying a DNA sequence encoding a putative dipeptide transmembrane transporter (Chr II: 4462621-4463890, reverse strand) similar to *Schizosaccharomyces cryophilus* EPY52281.1. RNA-Seq data of this region are shown. (C) Amino- and carboxyl-terminal expansions (forward strand in Chr II: 87554-87737 and Chr II: 89014-89176, respectively) highlighted by AnABlast analysis in the pseudogene SPBPB10D8.03 (arrows). The concatenated amino acid sequence of the different reading frame regions of this pseudogene, including carboxyl- and amino-terminal AnaBlast expansions, generated a protein 51% identical to a phthalate transporter of *S. cryophilus*. (D) AnABlast peaks (Chr I: 2955377-2955222 and Chr I: 29555220-29554983 respectively) (arrows) suggest changes in exons annotation in the pseudogene SPAPB24D3.05c (Chr I: 2955350-2955194 and Chr I: 2955191-2954983, respectively). Concatenated amino acid sequence of the reading frames predicted by AnABlast profiles produce a hypothetical pseudogene protein 65% identical to glyoxalase bacterial proteins. Pombase annotations and colour codes for reading frames of the analysed genomic intervals are shown. This figure is available in black and white in print and in colour at *DNA Research* online.

Interestingly, all the selected profiles, except one, were located in intergenic regions, suggesting that in general, *S. pombe* introns do not usually contain coding sequences. The only intronic peak was located in the first intron of the SPAC22F3.11c gene, *snu23* (Fig. 4A). RNA-Seq profiles, the identification of zinc-finger motifs, and its high identity to *Schizosaccharomyces sps* genes (Supplementary Table S1A) indicate that the sequence identified by AnABlast in this annotated intron is in fact a coding sequence of the *snu23* gene (Fig. 4A). A similar suggestion was made from *S. pombe* transcriptome analysis.^19^

### 3.3. AnABlast peaks highlighting pseudogenes

Annotated transposons and pseudogenes were not excluded for intergenic regions analysis. Accordingly, a number of transposons and all 29 annotated pseudogenes were detected in AnABlast profiles (see examples in Supplementary Fig. S1). In addition, AnABlast peaks uncovered a new pseudogene (Fig. 4B). Reading frames in this pseudogene contain numerous stop codons and at least five frameshifts, a complex structure that makes its identification by conventional methods difficult (see Fig. 4B). BLAST analysis of predicted amino acid sequences indicated that this new pseudogene encodes a putative dipeptide transmembrane transporter protein (Supplementary Table S1A). A second new pseudogene uncovered within rearranged DNA regions will be described later.

Detailed analysis of AnABlast profiles generated in annotated pseudogenes suggested a number of corrections to their genomic annotations. Two additional coding domains (in different reading frames) flanking the SPBPB10D8.03 pseudogene suggest carboxyl- and amino-terminal expansions in the annotated region (Fig. 4C and Supplementary Table S1A). Another correction is suggested in the two annotated exons of the SPAPB24D3.05c pseudogene. AnABlast also identified two regions separated by a single frameshift, although region intervals and the reading frame for the first region differ from those in the annotated exons (Fig. 4D and Supplementary Table S1A). Aside corrections, AnABlast peaks matching Ribo-Seq data profiles in pseudogenes suggest that at least two of them could be reassigned as coding genes (Supplementary Fig. S2).

From these results, we conclude that our *in silico* strategy efficiently highlights coding sequences in actual genes (Fig. 3) as well as in pseudogenes (Fig. 4). Since part of the pseudogenization process includes loss of initiating methionine, nonsense, and frameshift mutations, AnABlast might be useful to identify new genes, but also evolutionary degenerated coding sequences that escape to conventional searches.

### 3.4. Identification of new genes

The *S. pombe* genome is extremely well annotated.^16,20^ Thus, a stringent test for AnABlast is the identification of new genes in the genome of this model organism. To this end, we initially looked for AnABlast peaks containing ORFs, and 18 of them were selected.

**Figure 5.**
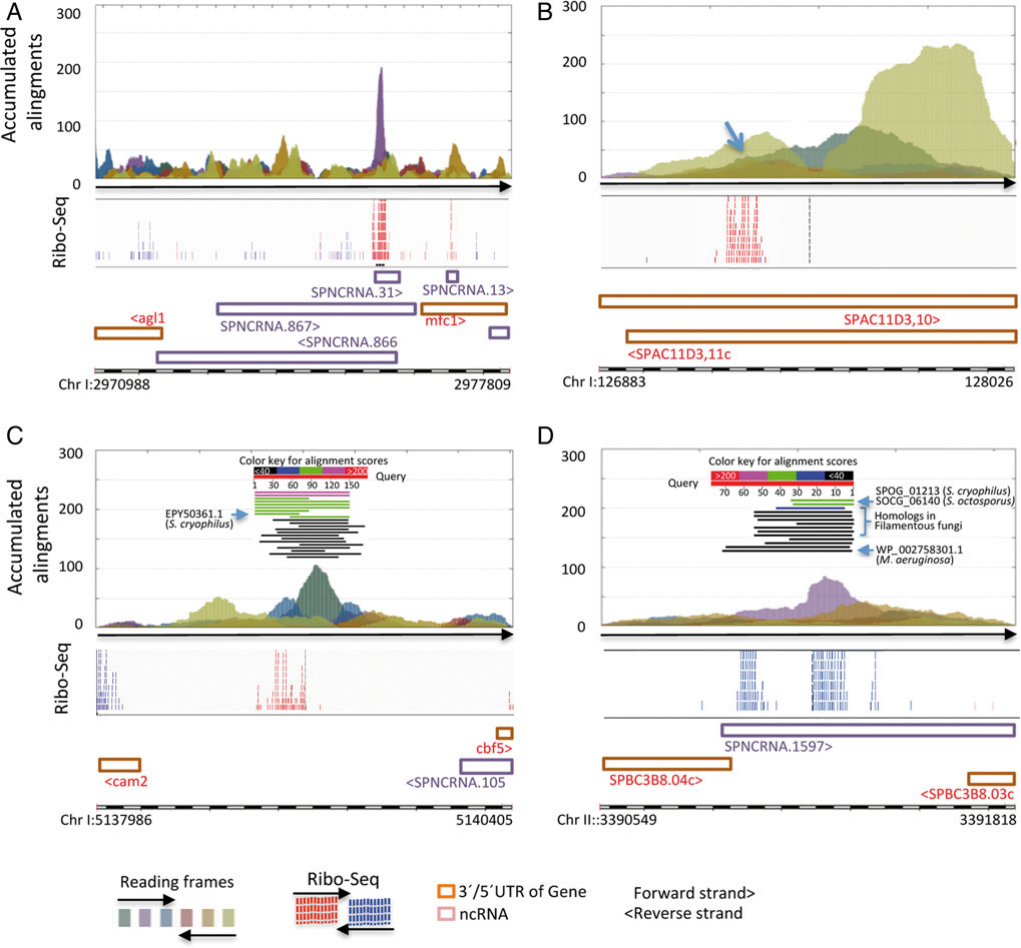
AnABlast profiles (accumulated alignments) identifying putative new genes. (A) AnaBlast peak at Chr I: 2975642-2975772 (forward strand) and (B) peak at Chr I: 127230-127316 (forward strand) (arrow) encode small peptides with no significant similarity to known proteins in databases. (C) Peak at Chr I: 5139023-5139195 (forward strand) identify a coding sequence similar to the SPOG_01629 protein of *Schizosaccharomyces cryophilus*. (D) Peak at Chr II: 3391178-3391413 (Chr II: 3391181-3391414, forward strand), uncovering a coding sequences with different degrees of similarity to protein SPOG_01213 from *S. cryophilus* and SOCG_06140 from *Schizosaccharomyces octosporus*, an hypothetical protein from a large number of filamentous fungi, and another one from the fresh water cyanobacteria *Microcystis aeruginosa*. (Schematic BLAST results are included). Ribo-Seq data, Pombase annotations, and colour codes for reading frames of the analysed genomic intervals are shown. This figure is available in black and white in print and in colour at *DNA Research* online.

Pfam and BLAST searches found that three of the selected ORFs uncovered new copies of annotated genes (Supplementary Fig. S3). Importantly, four other ORF-containing AnABlast peaks identified putative new protein-coding genes, experimentally validated by Ribo-Seq expression patterns^18^ (Fig. 5 and Supplementary Table S1B).

The first novel gene partially overlaps the predicted poly(A)-bearing non-coding RNA SPNCRNA.31. Both the AnABlast ORF (42 amino acid length) and the annotated non-coding RNA are found in the forward strand, with the Ribo-Seq profile confirming the translation of the ORF (Fig. 5A).

The second new gene contains a short 28 residues ORF that is highly expressed (Fig. 5B). The corresponding AnABlast peak further extends the carboxyl-terminal end of this ORF (see in Fig. 5B), suggesting that this extremely short gene could be originated through a carboxyl-terminal subtraction from a longer ancient one.^26^ Polypeptides encoded by these two new genes lack significant homologs or motifs in databases.

The other two AnABlast ORFs, in addition to being translated, encode polypeptides that share significant similarity to known proteins. A main AnABlast peak, flanked by two others in different reading frames, identifies one of these new genes (see in Fig. 5C). Concatenated amino acid sequences from the three peaks render a 178-residue protein with a homologue in *Schizosaccharomyces octosporus* and eight in *Schizosaccharomyces cryophilus*, ranging in length from 102 to 190 residues (Supplementary Table S1B). RNA expression profile (see Ribo-Seq pattern in Fig. 5C) and its homology to the short EPY50361.1 *S. cryophilus* variant (see alignments in Fig. 5C) suggest that only the 56 residues ORF encoded by the first AnABlast peak is an active gene. Thus, the proposed *S. pombe* gene (and its equivalent in *Schizosaccharomyces cryophilus*) likely resulted from premature nonsense mutations, generating a fossil carboxyl-terminal sequence that in the remaining related proteins is still active.

Another AnABlast ORF encodes a 77-residue polypeptide sharing 29% identity (52% similarity) along the entire sequence of the WP_002758301.1 protein from *Microcystis aeruginosa*, an evolutionary distant freshwater cyanobacteria (Fig. 5D). Identity increases to 35% with homolog proteins found in filamentous fungi, but homology length decreases to the first 52 residues in these closer microorganisms. Identity with homolog sequences found in *S. cryophilus* and *S. octosporus* rises to 76% but is limited to 31 amino-terminal residues. Since RNA expression is also observed at this 31 amino acids interval, we conclude that this short region is an active coding sequence in fission yeasts. According to BLAST alignments (Fig. 5D), premature nonsense mutations could also result in the progressive carboxyl-terminal subtractions of this coding sequence over evolution.^26^ However, in *S. pombe*, no stop codon is found at the 3′ end position of the DNA sequence coding for the 31-residue domain (see in Supplementary Table S1). In fact, RNA-Seq data suggest the existence of at least two exons in this region (see Fig. 5D). Thus, in this particular case, subtraction likely raised in the *S. pombe* genome by the creation of a new splice site.^27,28^

We cannot rule out the existence of new genes in the remaining ORFs found by AnABlast search. However, neither significant translation levels nor sequence/motif conservation support their actual gene status (see examples in Supplementary Fig. S4).

The *in silico* identification of new genes is particularly useful for genome annotations. To test the potential use of AnABlast as a gene-finger tool in other organisms, we analysed the Chromosome II:0-5 Mb interval of the *C. elegans* genome. Annotated ORFs were efficiently highlighted in AnABlast profiles using the settings trained for the *S. pombe* genome. Furthermore, a detailed analysis of intergenic regions in this interval revealed that AnABlast profiles can also be useful to identify new genes in the nematode genome (Supplementary Fig. S5).

### 3.5. Ancestral gene subtractions

**Figure 6.**
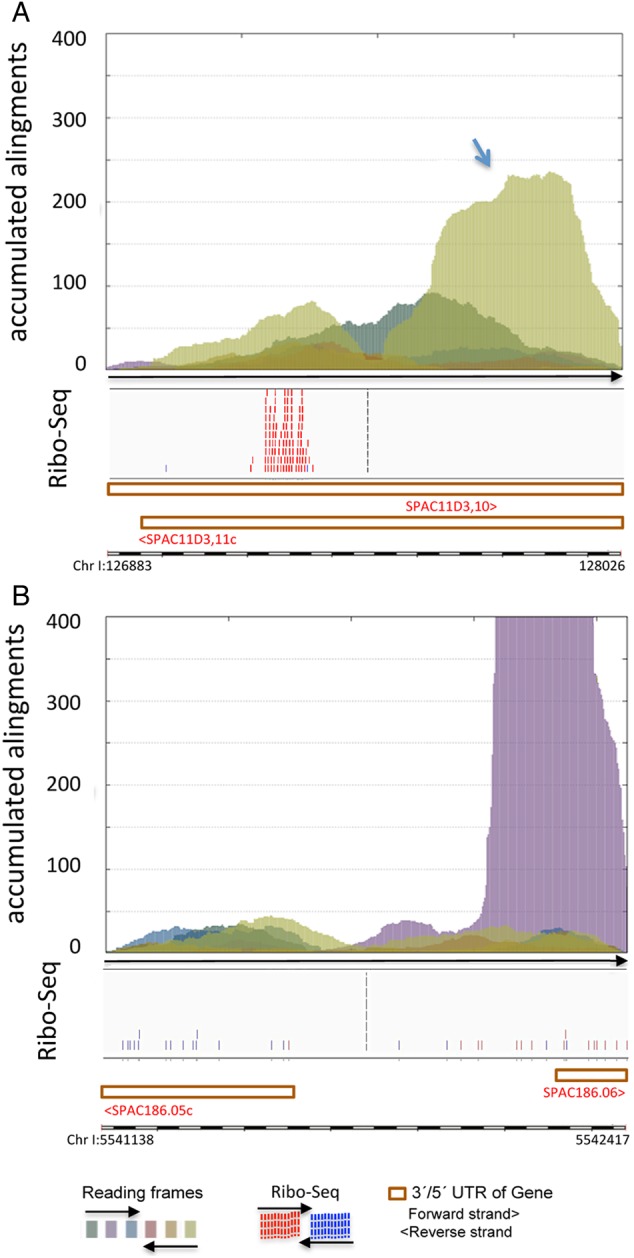
AnABlast peaks uncovering evolutionary carboxyl- and amino-terminal subtractions in present genes. (A) AnABlast peak (arrow) at Chr I: 127.165-128.049 (reverse strand) located in the 3′UTR of SPAC11D3.11c reveals a carboxyl-terminal subtraction of this gene. (B) Peak at Chr I: 5542072-5542417 (forward strand), partially overlapping the 5′ UTR of SPAC186.06, uncovers an evolutionary amino-terminal subtraction of the corresponding gene. Ribo-Seq data, Pombase annotations, and colour codes for reading frames of the analysed genomic intervals are shown. This figure is available in black and white in print and in colour at *DNA Research* online.

As shown above, our coding sequences search strategy can both find new genes and uncover their associated evolutionary history, revealing fossil-coding sequences that have being subtracted by changes in stop positions (Figs 5B and 3C) or in splice sites (Fig. 5D). To locate possible fossil coding sequences subtracted from present genes, we examined peaks located at the edge (exon boundaries) of AnABlast profiles at each inter-exon region. Peaks revealing amino acid sequences encoded in the same strand as adjacent exons were selected (putative ancient subtractions), and their corresponding amino acid sequences concatenated ignoring reading frameshifts and stop codons. Interestingly, BLAST searches identified two genes in which alignments to homologous proteins extended along the concatenated peptides, one by expanding the 5′ end coding DNA sequence and the other through a 3′ end extension (Fig. 6A and 4B, and Supplementary Table S1C).

Evolutionary changes in coding sequence length seem to be dynamic processes via shifts in the position of the start^29^ and the stop codons.^26^ Our findings indicate that AnABlast may be useful to identify subtractions occurred during evolution. Remarkably, AnABlast can identify long terminal changes which, according to previous studies,^26,29^ represent rare events among terminal subtractions and expansions. Since evolutionary expansions are part of present genes, AnABlast analysis of intergenic regions carried out in this study cannot uncover these evolutionary events.

### 3.6. Identification of gene fragments

**Figure 7.**
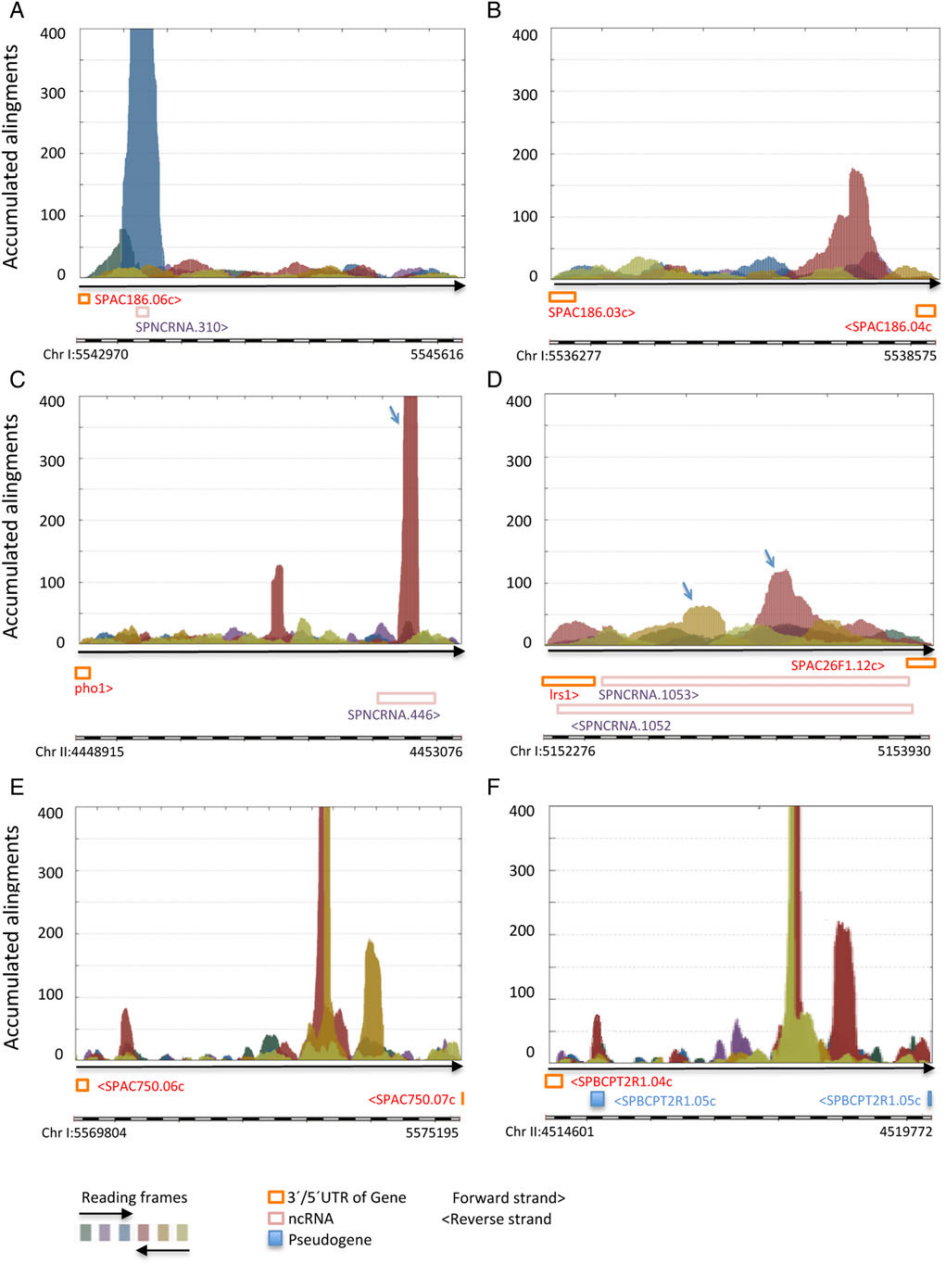
Gene fragments identified by AnABlast profiles. (A) Peak in the Chr I: 5543318-5543498 interval (forward strand) uncovers a fragment of Tf1 protein (*S. pombe* ref gb|AAA35339.1|, e-value: 2e^−15^). (B) Peak in the Chr I: 5536277-5538575 interval (reverse strand) encodes a polypeptide sequence belonging to a MIP water channel (*S. pombe* ref|NP_592788.1|, e-value: 5e^−12^). (C) Main AnABlast peak (arrow) at Chr II: 4452616-4452379 encodes a chimeric fragment of amino acid permeases (Chr II: 4452616-4452443, *S. pombe* ref|NP_596849.1|. e-value: 2e^−20^) fused in frame to another one sharing significant identity to bacterial transposases (Chr II: 4452444-4452379, *Desulfobacter postgatei* ref|WP_004074224.1|, 73% identity). (D) Peaks (arrows) at the Chr I: 5153233-5153041 and Chr I: E 5153042-5152866 intervals (reverse strand) encode fragments of cell surface glycoproteins (*S. pombe* ref|NP_588570.2|, e-values of concatenated sequence: 3e^−72^). (E and F) Genomic subtelomeric regions (Chr I: 5569804-5575195 and Chr II: 4514601-4519772, respectively) showing similar AnABlast profiles highlighting fragments of RecQ type DNA helicase coding sequences (*Schizosaccharomyces pombe* ref|NP_595040.1|, e-value: 0,0) and DUF999 family proteins (SPAC212.04c, e-value: 0,0). Pombase annotations and colour codes for reading frames of the analysed genomic intervals are shown. This figure is available in black and white in print and in colour at *DNA Research* online.

The vast majority of the selected AnABlast profiles identified single peak regions without putative ORFs (63%, excluding pseudogenes and transposons). Since AnABlast identifies coding sequences independently of ORFs, most of these peaks could represent gene fragments arisen from mutations and/or genome rearrangements. Accordingly, careful BLAST and Pfam analysis of predicted amino acid sequences in some of these peaks revealed fragments of tf1-retrotransposon (Fig. 7A), of a MIP water channel protein (Fig. 7B), and of an amino acid permease (Fig. 7C). Interestingly, this later peak includes a possible fossil fragment of a bacterial transposase, fused to the amino acid permease sequence in the same reading frame (Supplementary Table S1D). This finding casts light on how random combinatorial protein domains, including exogenous DNA, may contribute to the emergence of new traits during evolution.

Our search strategy also identified rearranged fragments of the same gene. Two adjacent genomic intervals coding for partial sequences of a predicted surface glycoprotein (in different reading frames) (Fig. 7D), or up to three different subtelomeric regions encoding adjacent fragments of the RecA protein (encoded by the *tlh1*/*tlh2* genes) provide good examples of rearranged genes. As shown by AnABlast profiles, at least two regions containing *tlh1*/*tlh2* DNA fragments located in subtelomeric regions of Chromosomes I and II, respectively, harbour identical rearranged structures (see Fig. 7E and F). A fragment of the SPAC212.04c gene (DUF999 family protein 1) can also be observed in both subtelomeric intervals (see in Fig. 7), albeit only in the case of Chromosome II this gene fragment is annotated as pseudogene (SPBCPT2R1.05c). According to AnABlast peaks, this pseudogene should be also annotated as new pseudogene in the equivalent Chromosome I subtelomeric region.

Interestingly, the identical genomic gene fragments pattern revealed by AnABlast profiles in both subtelomeric intervals suggests that evolutionary events yielding such fragments likely occurred earlier than their translocations to the subtelomeric region. However, we cannot rule out that occasionally, these AnaBlast results could point to possible genome assembly errors rather than rearranged fragments and/or missing annotations.

### 3.7. Fossil-coding sequences

As described above, BLAST analysis of a particular AnABlast peak identified a possible fossil transposase sequence, perhaps of bacterial origin (see in Fig. 7C). This observation led us to investigate whether other AnABlast peaks could identify fossil sequences^30^ transferred from other genomes.

**Figure 8.**
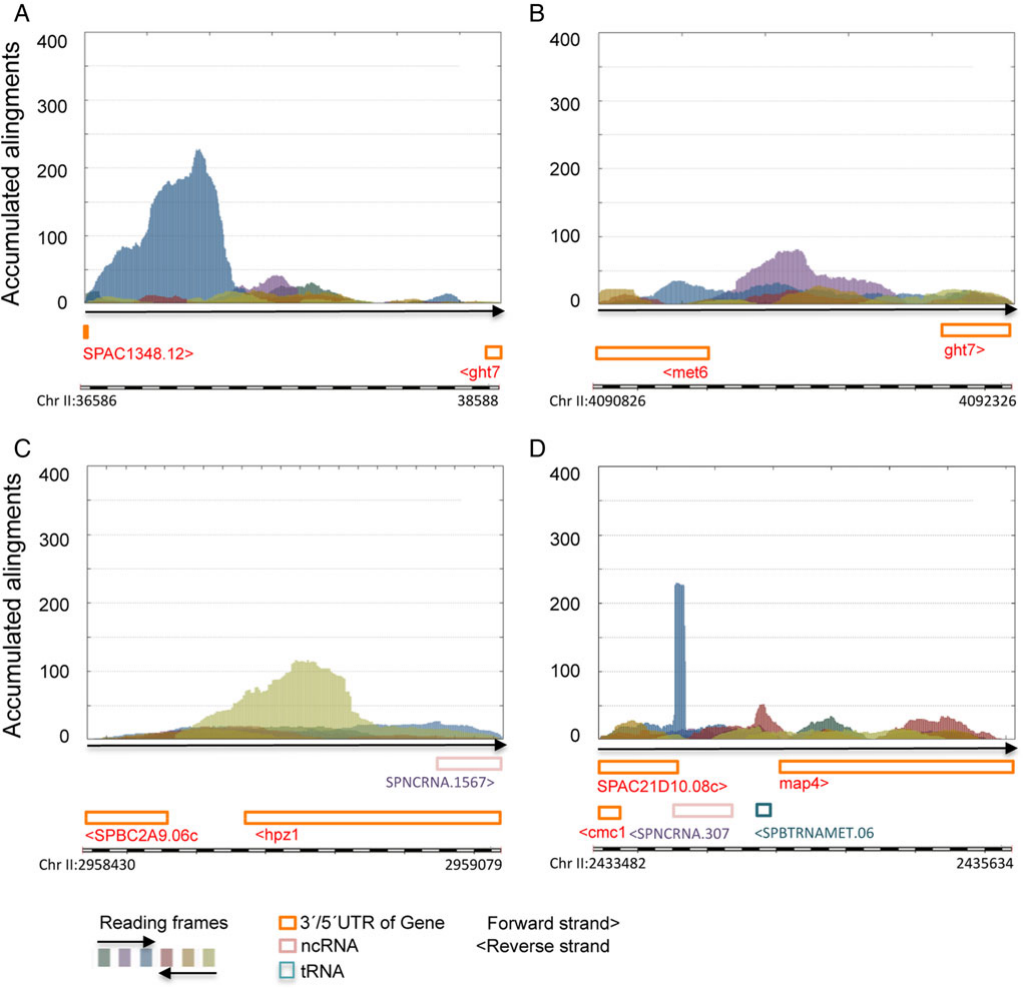
Fossil-coding sequences predicted by AnABlast search. (A) AnABlast peak (Chr II: 36890-37298, forward strand) encodes a fragment of mitochondrial maturase protein 2 (fission yeast mitochondrion. Sequence ID: pir|S78197|. *E*-value: 5e^−56^). (B) Peak at Chr II: 4091456-4091554 (forward strand) codes for a polypeptide 58% identical to a domain of bacterial transposases (*Bacillus mycoides* ref: gb|KFN12866.1|). (C) Peak at 2958676-2958843 (reverse strand) encodes a partial sequence of bacterial trehalose synthase (*Streptomyces chartreusis*, ref|WP_010033287.1| *e*-value: 1e^−17^). (D) Peak at Chr II: 22433875-22433935 (forward strand) identifies a small peptide domain common to numerous cytochrome c oxidases (*e*-value for *Hylarana sp*: 1e^−07^). Pombase annotations and colour codes for reading frames of the analysed genomic intervals are shown. This figure is available in black and white in print and in colour at *DNA Research* online.

Importantly, AnABlast identified a coding sequence containing a protein domain of the mitochondrial maturase protein-2 (SPMIT.03 mitochondrial gene), indicating that this genome fragment has a mitochondrial origin (Fig. 8A). Furthermore, additional transposase and trehalose synthase fragments (Fig. 8B and C, respectively) point to a possible horizontal transfer from bacteria as an evolutionary source for these intergenic sequences.^31^ A short region encoding amino acid sequences highly similar to cytochrome c oxidase subunit III was also identified (Fig. 8D).

### 3.8. Conclusion

Here we report that low-stringency alignments accumulation in predicted amino acid sequences may discern coding from non-coding DNA, thereby providing a new *in silico* strategy (AnABlast) to reveal present and ancient coding regions in sequenced DNA. We have shown that the identification of rearranged and fossil sequences by AnABlast may be helpful to better understand the evolutionary history of the *S. pombe* genome. This property, together with the ability to uncover novel genes and pseudogenes, suggests that AnABlast provides a new strategy of general interest for the genome-wide search of present and fossil sequences in any genome.

## Authors' contribution

J.J. and A.P.P. conceived the study and designed the experiments. A.P.P. developed the computer programme. A.P.P. and M.G. generated AnABlast profiles. C.D.S.D. and J.M. performed Ribo-Seq experiments. J.J. did the analyses of AnABlast peaks and wrote the manuscript. J.J., C.D.S.D., M.G., J.M., and A.P.P. critically read and approved the manuscript.

## Supplementary data

Supplementary data are available at www.dnaresearch.oxfordjournals.org.

## Funding

This research was supported by the Ministry of Economy and Competitiveness of the Spanish Government grant BFU2013-46923-P (J.J.) and by Biotechnology and Biological Sciences Research Council (UK) research grant BB/J007153/1 (J.M.). Authors declare no competing interest. Funding to pay the Open Access publication charges for this article was provided by the Ministry of Economy and Competitiveness of the Spanish Government.

## Supplementary Material

Supplementary Data
