# Supplementary material for: AnABlast: a new in silico strategy for the genome-wide search of novel genes and fossil regions
Source: DNA Res. 2015 Oct 21;22(6):439–49. doi: 10.1093/dnares/dsv025 (PMC4675712; doi:10.1093/dnares/dsv025)
Supplement: Supplementary Data [file supp_22_6_439__index.html]

AnABlast: a new in silico strategy for the genome-wide search of novel genes and fossil regions — Supplementary Data 

# AnABlast: a new *in silico* strategy for the genome-wide search of novel genes and fossil regions

## Supplementary Data

Supplementary Data

- Supplementary Data - Pdf file
