# Supplementary material for: AnABlast: a new in silico strategy for the genome-wide search of novel genes and fossil regions
Source: DNA Res. 2015 Oct 21;22(6):439–49. doi: 10.1093/dnares/dsv025 (PMC4675712; doi:10.1093/dnares/dsv025)
Supplement: Supplementary Data [file supp_dsv025_dsv025supp.pdf]

Supplementary data

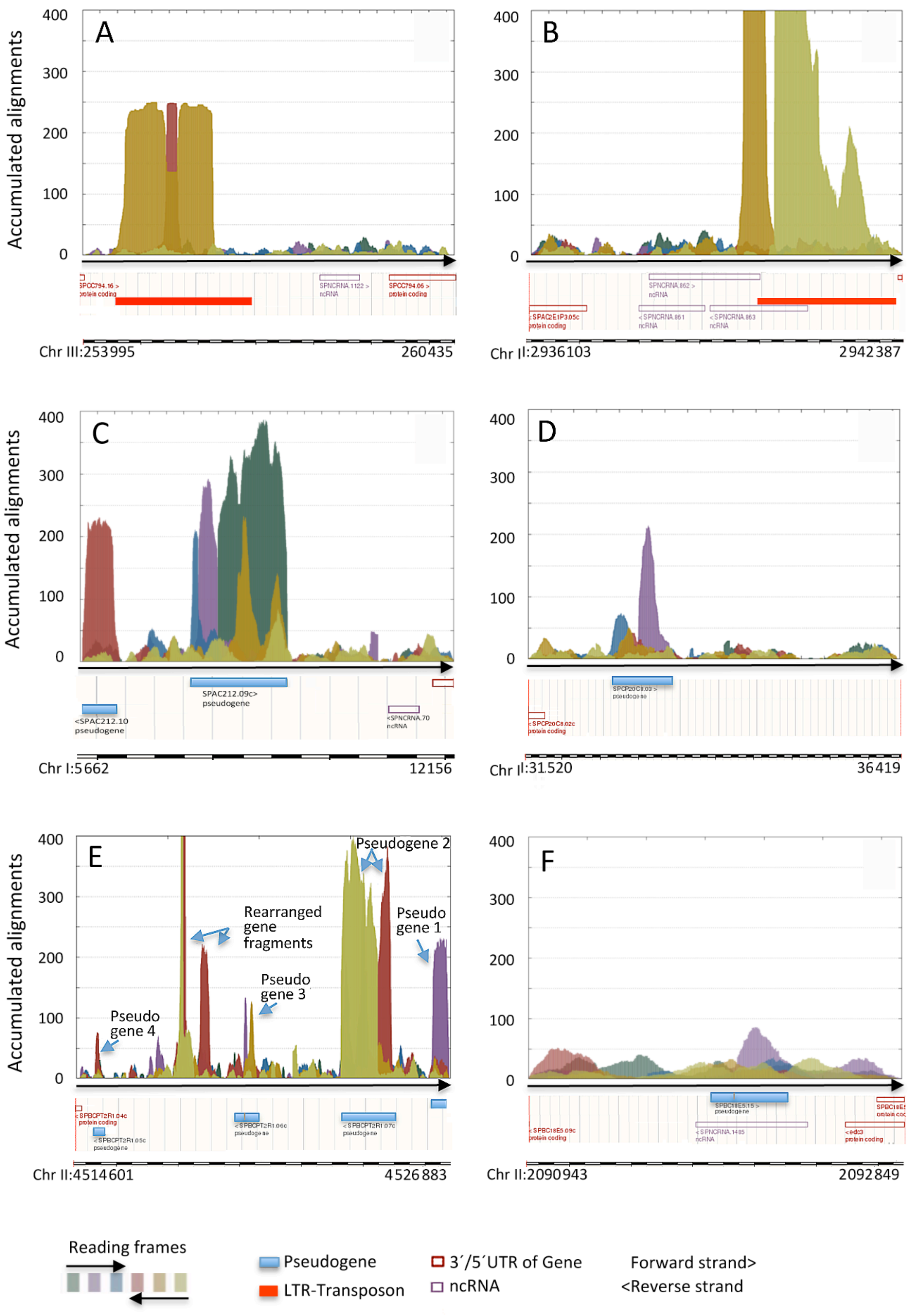

Supplementary Figure S1. AnaBlast Profiles annotated transposons and pseudogenes. A) and B) Peaks matching annotated transposons. C), D), E) and F) Annotated pseudogenes highlighted by AnABlast peaks in the indicated intergenic regions. Pombase annotations and colour codes for reading frames of the analysed genomic intervals are shown.

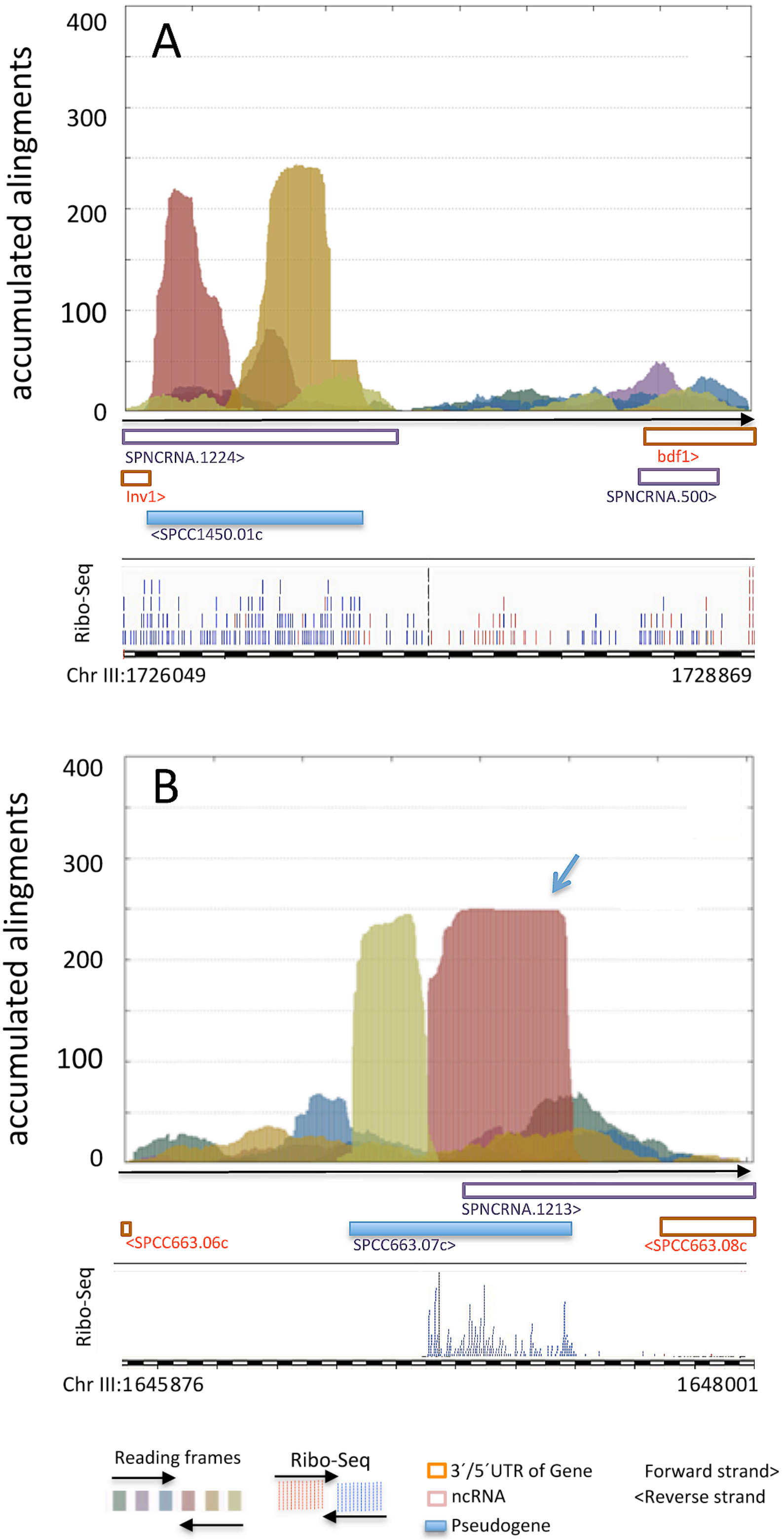

Supplementary Figure S2. Annotated pseudogenes proposed as coding genes. A) Pseudogene SPCC191.12c yield two AnABlast peaks in different reading frames coding for bacterial alpha/beta-hydrolase. Provided that an intron links both coding regions, expression patterns suggest that this pseudogene could be a coding gene. B) The SPCC663.07c pseudogene (coding for a short chain dehydrogenase) generates two AnABlast separated peaks. The first one (arrow) matches a defined ORF (170 residues) which is expressed. Ribo-Seq data (translation), Pombase annotations and colour codes for reading frames of the analysed genomic intervals are shown.

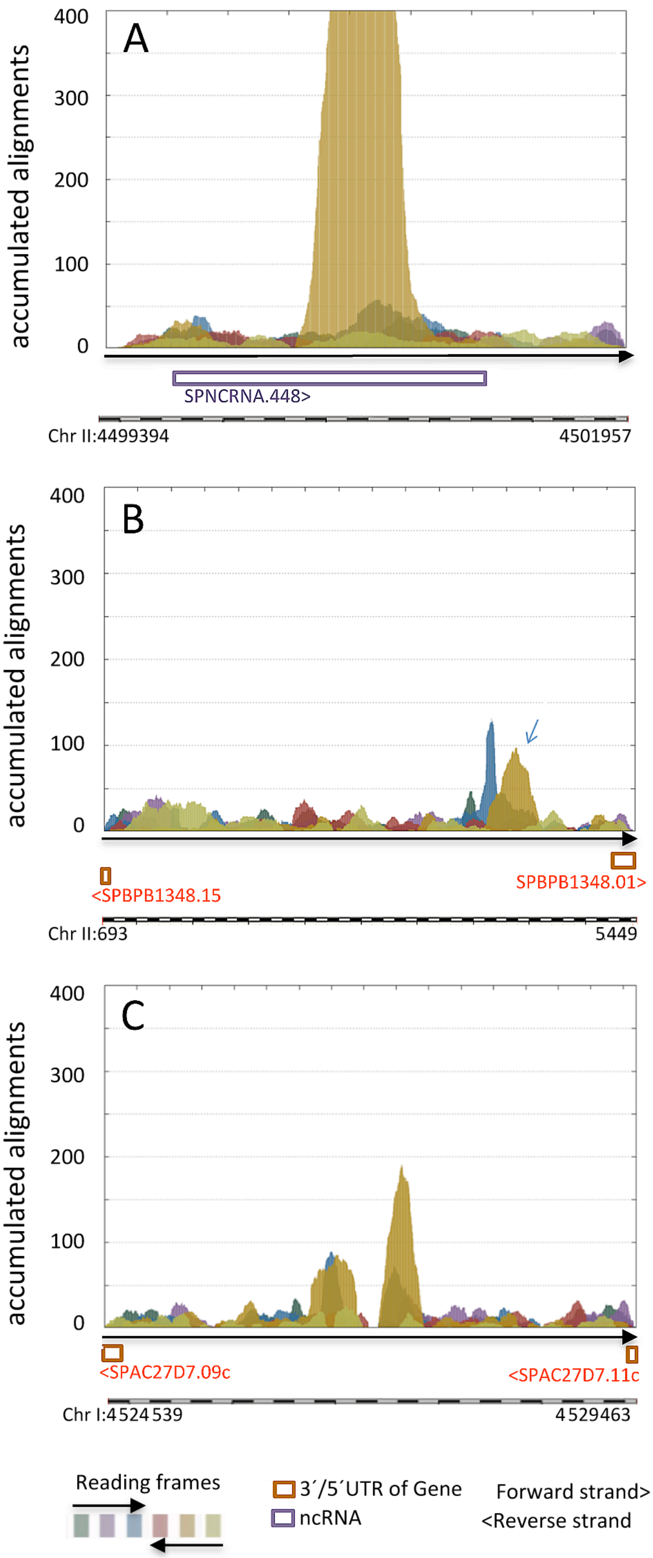

Supplementary Figure S3. New copies of annotated genes. A) AnaBlast peak at Chr II: 4500790-4500352 interval (reverse strand) predicts a new copy of annotated SPBC1348.04, SPAC750.03c, SPAC977.03 genes (coding for predicted methyltransferase). B) Peak at Chr II: 4114-4473 (reverse strand) (arrow) identifies another copy of genes SPBCPT2R1.02 and SPAC212.02 (sequence orphan). C) Peak at Chr I: 4527576-4526425 (reverse strand) corresponds to a new copy of SPAC27D7.9c and SPAC27D7.11c (*but2*). Pombase annotations and colour codes for reading frames of the analysed genomic intervals are shown.

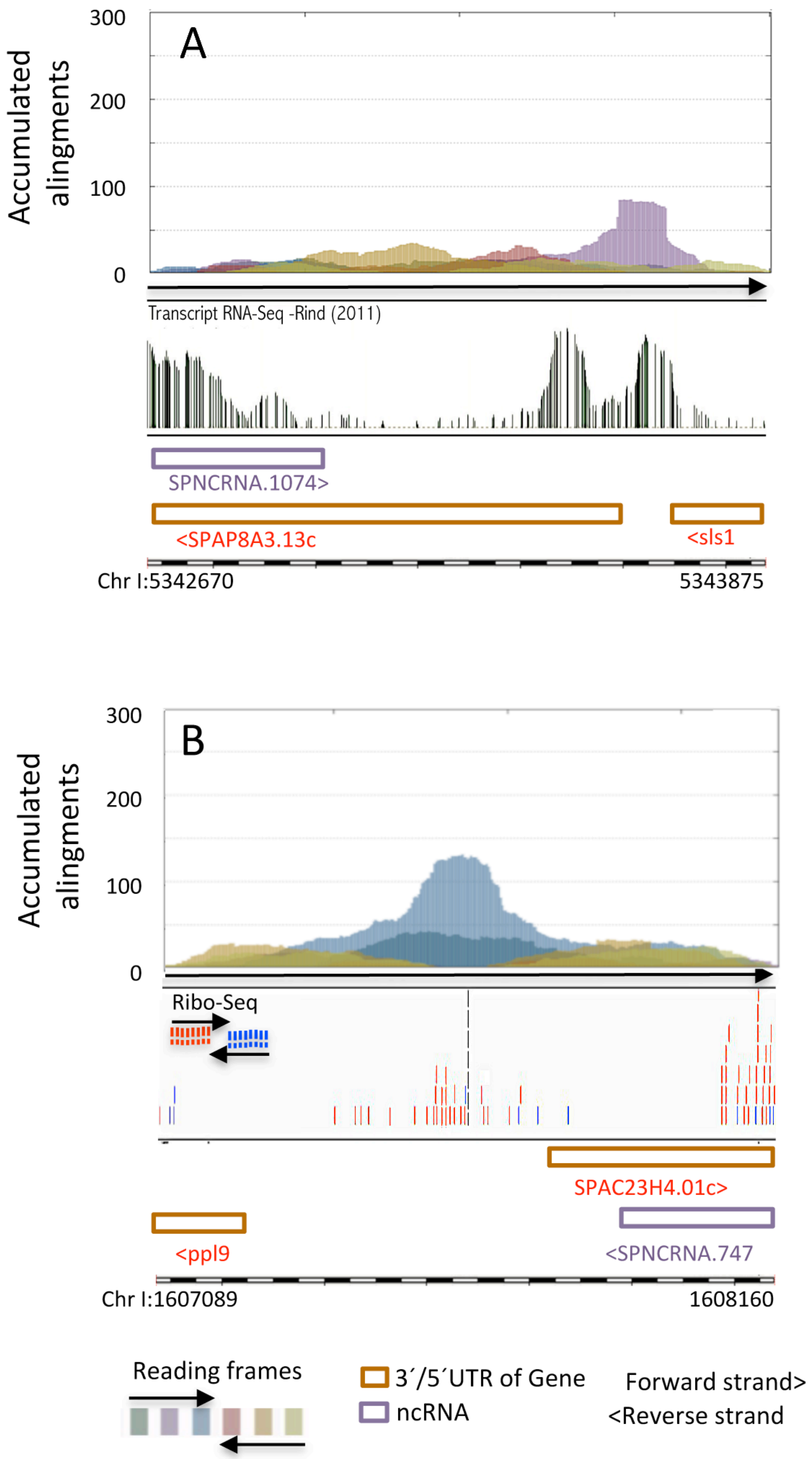

Supplementary Figure S4. AnABlast profiles of ancient coding regions and RNA expression. A) AnaBlast peak at Chr II: 4500790-4500352 interval (reverse strand). RNA-Seq data from quiescent cells are shown. B) Peak at Chr II: 4114-4473 (reverse strand) (arrow). Ribo-Seq data from meiotic cells are shown. Pombase annotations and colour codes for reading frames of the analysed genomic intervals are indicated.

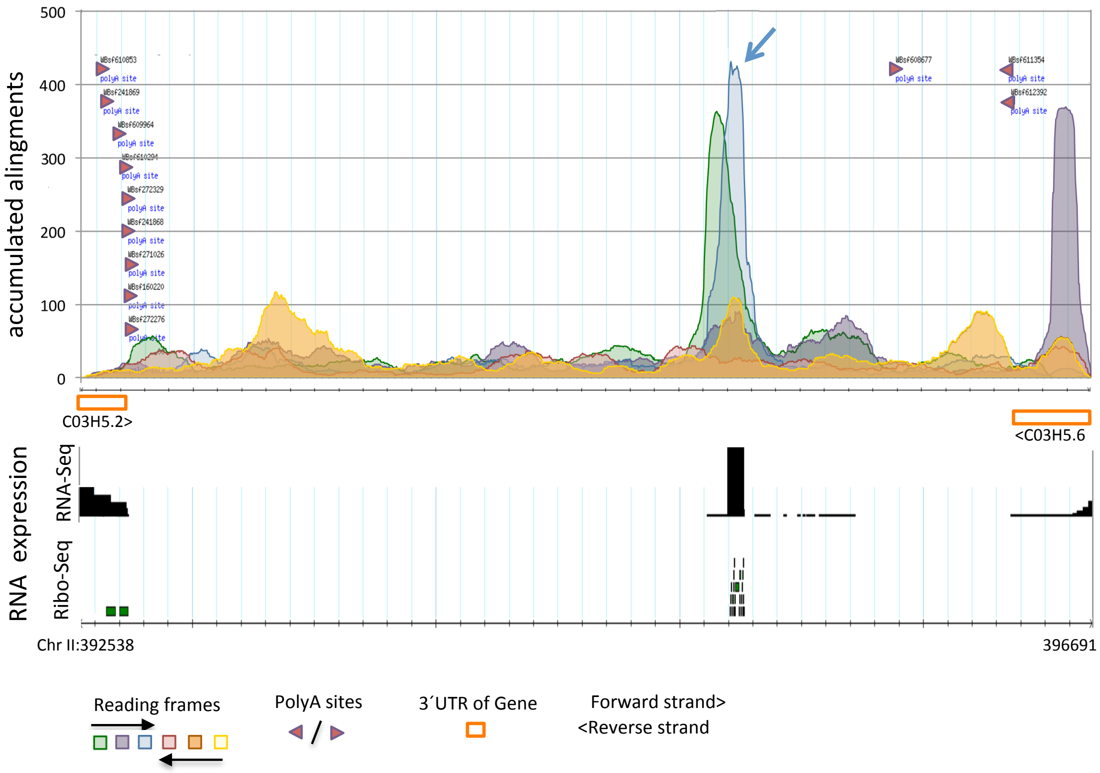

Supplementary Figure S5. AnABlast profiles of the *C. elegans* Chr II: 392.538-396.691 intergenic region. According to WormBase genomic browser, this region is flanked by the protein-coding genes C03H5.2 (nstp-4) and C03H5.6. The indicated AnaBlast peak (arrow) perfectly matches with strong RNA-Seq and Ribo-Seq signals. This sequence highlighted by AnABlast contains a motif found in S26 ribosomal proteins. The RNASeq signal extends to the 5´ region which presents other AnaBlast peaks in a different reading frame, and to the 3’ end until a probable polyadenylation site.

Table S1. Summary of results from AnABlast analysis of all inter-exon regions annotated in the *S. pombe* genome.

A) Proposed corrections in Pombase genome annotations according to AnABlast predictions

**a) Chromosome: inter-exon interval**<br>**b) AnABlast peak interval/Coding strand**<br>**c) Comments from prediction**<br>**d) BLAST analysis (e-value) of predicted sequence**<br>**e) Fig. in main text**	**Predicted amino acid sequence from AnaBlast**
a) Chromosome I: 683034-683100<br>b) 683035-683099/Forward strand<br>c) Annotated intron in gene SPAC22F3.11c belong to the coding sequence. "C" at genome position 1:682997 should be deleted.<br>d) Zinc finger protein<br>e) Fig. 4A	Protein sequence (in **bold**, correction from AnABlast prediction)<br><br>MDRYNPPRRNGNSKKNEVVITGGRTQRIDFEKDVNKT<br>TILP**AGASVGRRGRGAGWYCEACNETYKDSLSWLD**<br>**HLN**STQHLRKTRTVIIEKRATLEEVKERMEYWRRQLLE<br>PEKGSEEYSLKERVERYHQELEAKKLRRKQKKVNKEK<br>NSPRLVGENTELAAIMGISSFGSTNL
a) Chromosome II: 4461701-4466752<br>b) 4462621-4463890/Reverse strand<br>c) New Pseudogene<br>d) Dipeptide transmembrane transporter (*Schizosaccharomyces pombe* 972h-)<br>Sequence ID: ref|NP_587688.1| ($8e^{-39}$)<br>e) Fig. 4B	Peaks 1-6:<br>MVEHLDKTPLLYAALYDMKQDTHIDIYTYSSMTILCGS<br>YLVAQYPAALLM<br><br>LWSALVCLMAACRNGPFLLGLRFLTXIFKAPITPAFINIT<br><br>MITNGIGVFSTLIIKGLGYNQLRTTVLSMPSGAIEIA<br><br>CAXFLAAVFIRFLKNSRLLAVNFFNCLTRSGCIXIWKIP<br>ASYPWGRLVGVWFIVWLPAAN<br><br>TFLFSLIVTNIAGCTKKRLLVPLHLLFIYLVTLFRRNYLN<br>GHTPEYIEKKAVTSATAFTFYLLGNIVSPQLLKWSYTGI<br>YXRNLSNDCFSCVIVVT<br><br>SLLINVKNEEFLDQTDR
a) Chromosome II: 85431-91725<br>b) 87554-89176/Forward strand<br>c) Pseudogene SPBPB10D8.03 carboxi- and amino-terminal coding expansions.<br>d) Phthalate transporter (*Schizosaccharomyces cryophilus* OY26)gb|EPY52281.1|Length: 585<br>($6e^{-162}$)<br>e) Fig. 4C	Amino-terminal expansion (87554-87737)<br>M L E L G E P X E F S K F F W K R R K L D P N S I T T K<br>K S I F D D D P N T L A A F K P S P Y Y E S R H R I D P F F<br>R W I W<br><br>Carboxi-terminal expansion (89014-89176)<br>K I F Y V S L N K Y R D R K W N A M T E N E K N E F G<br>N N Q R H R N A S F G F S I R P L T L E F E Y M I F
a) Chromosome I: 2954824-2956065<br>b) Peak 1:2955378-2955222/Reverse strand<br>Peak 2:29555223-29554983/Reverse strand<br>c) Pseudogene SPAPB24D3.05c. Error in annotated first exon<br>d) Glyoxalase (*Rhizobium leguminosarum bv. trifolii* WSM2304)<br>($5e^{-39}$)<br>e) Fig. 4D	Peak 1<br>M E K D I I X I D R F H H I V F T V Q D V E A T C E F Y<br>E R T L G M S V I A F G N N R K A L G F G H K K<br><br>Peak 2<br>I N L H Q K G N E I D P K S H I L T P G S N D I C L I A K<br>T P L E Q V I A H L K K Q N V L I E E G P I T R A G A E<br>G V I L S I Y F R D P D E N L I E V S N Y I

B) Putative new genes

**a) Chromosome: Inter-exon interval**<br>**b) AnABlast peak interval/Coding strand**<br>**c) BLAST analysis (e-value) of predicted sequence**<br>**d) Fig. in main text**	**Predicted amino acid sequence from AnaBlast**
a) Chromosome I: 2970988-2977809<br>b) 2975642-2975772/Forward strand<br>c) No significant similarity found<br>d) Fig. 5A	Met T T G K P Q S F E K M R T P F P G R S K<br>A K G P Q S D I I P S A P P N T P V T E H Stop
a) Chromosome I: 126883-128026<br>b) 127230-127316/Forward strand<br>c) No significant similarity found<br>d) Fig. 5B	M S F S K F H C G K L M S L K L A D L R T M<br>F E A L E K Stop
a) Chromosome I: 5137986-5140405<br>b) 5139023 -5139195/Forward strand<br>c) Hypothetical protein SPOG_01629 (*Schizosaccharomyces cryophilus* OY26) ($3e^{-13}$)<br>d) Fig. 5C	First exon:<br>Met K I D K A I F T N Q F G V P L P S D N P Y<br>V A L H D F V Y D L E V A I P E D E F E A F K<br>E Q L A N P R N S C M Stop<br>Second exon:<br>C E V R E S I S K K D S I F Q T Y E M L Q E L<br>K D G Q S S L R E D L N H L S H G Q N V L K<br>K N Met V Y L N G T F E C I S N V I R A N N<br>Q I L M L E F G K S N R K<br>Third exon:<br>P D T D A R I W K V E S E V N E L K V S A Q<br>R S E E I L N Y K S A K Q M N S N L S T I Y R<br>V L P L I K I F L M N I R N C L N Stop
a) Chromosome II: 3390549-3391818<br>b) 3391178-3391413/Forward strand<br>c) Hypothetical protein SPOG_01213 (*Schizosaccharomyces cryophilus* OY26) ($1e^{-11}$)<br>d) Fig. 5D	M F Y K N Y A R P L G K V T L F A L A T Y Y<br>G L E I V W W K L D A S E Q E A I K N S K L<br>L I C E S S F S L L T F R R I T E F R E C E I K<br>T R D L Y D P E I Stop

C) Carboxi- and amino-terminal subtractions

**a) Chromosome: Inter-exon interval**<br>**b) AnABlast peak interval/Coding strand**<br>**c) Comments from prediction**<br>**d) BLAST analysis (e-value) of predicted sequence**<br>**e) Fig. in main text**	**Predicted amino acid sequence from AnaBlast**
a) Chromosome I: 126883-128026<br>b) 127165-128049/Reverse strand<br>c) Located in the 3´UTR of SPAC11D3.11c: zinc finger protein<br>d) Concatenated sequences: Fungal zinc cluster transcription factor (*Schizosaccharomyces cryophilus* OY26) ref gb|EPY50439.1| (0.0)<br>e) Fig. 6A	Protein sequence of annotated SPAC11D3.11c gene<br>M F K E Y D S R K I R H P H S Y V K A L E T<br>R L A G L E A F W K R V K Y A P V N E K L E<br>L L K T I S F N D H L S P D I S V S K T D T T F<br>E F P V S L D I R G P N T I A F Y G P T N V Y<br>G P P L T P S S P E T P S F P P Q N P S F S P L<br>I T D C L K L F F K W Q Y P Q F L F I N R E A<br>F L V D Y Y Y R Y H E G R Y C S E H L L Y A<br>M C A I G S R M S V D P N I A A L A K N F Y<br>Q I A W N K I I E Y G L G K S H I T S I Q C L L<br>C L G Y F N I G M G N T S L G W M L S G M<br>A F R M G Q D L G F Q L N P R N W S V N D<br>H P V V S P A D A A V R S R I Y W G S Y V T<br>D I F I S F V L G R P T T L K K S D T S I P D S<br>E S L P D F D G V N E Y R V N N A L L L K E<br>Y L C I Q S S V Y<br><br>Predicted carboxi-terminal substraction<br>M Y T I L C L L V D L S N V A D A I L L N V F<br>A P I S M P Y G A D Y M L Q N L G K Y N L E<br>L A K W H L K L P S E L R W K K S E L R K F<br>G Q N P D L S V V C L Y Y N L I K I C L N R P<br>F L S S K E V I S N D M T P K L I C Q D S I E<br>D I E L L I K Y R N D A F G I N S V S F V L V<br>Y A A I V S C S V L L M L W N D E D P I L N<br>R Q V I E T K L N F F L I V L V K A S K V W D<br>L A T K S I H L I K S S L L S K G V A F T D S<br>F S L K S N S E G L N V N N S N S S I A H E S<br>A N I P D I L D P S T K T F E L D I T S Q G L Q<br>T L Y G G P P I L M T S A S H S E I W K N S L<br>F S M F N Q P N D S E D G N N R L Y E Q
a) Chromosome I: 5541138-5542417<br>b) 5542072-5542417/Forward strand<br>c) Intergenic location, partially overlapping the 5' UTR of SPAC186.06. No significant similarity found<br>d) Concatenated sequences:<br>1) Phenazine biosynthesis-like protein (*Escherichia coli*) ref|WP_000804359.1| ($8e^{-98}$)<br>2) RNA-mediated gene silencing enhancer protein (*Schizosaccharomyces pombe* 972h-) ref|NP_001018772.1| ($3.1e^{-66}$)<br>e) Fig. 6B	Protein sequence of annotated SPAC186.06 gene<br>Met S I K L G Y L F K L P S Y K T S S I S P E V<br>I V D L E K F L N L C E G S C S Q C N T P F C<br>V D V G P R N A I I Q L P N G A D V L N L N P<br>N F Q A I F E C C S K N S L T G V Q I F G M Y<br>N D G T Y E L R S F C P V H G V N E D P A N<br>G S G A G S V G V F F A L N N P S I I S S D F<br>A H L L F N Q G K I L G R N A L I R V A I K L<br>S A N G L Y D I H V G G G S K I C I S G T A E<br>I Stop<br><br>Predicted amino-terminal subtraction<br>Met A L Y P F R Q V D V F S T K G Y A G N P<br>L A V F L D A D G L N S T Q M X Q I A K W T<br>N L S E T T F I Q K P T I P G T D Y R I R I F T<br>P G C E L P F A G H P T I G S C F V L L D N K<br>S C S A K K N K V V Q Q C N S G L V E L T L<br>H N E D V N Q T W I S F Q I T E L

D) Fossil coding sequences

**a) Chromosome: Inter-exon interval**<br>**b) AnABlast peak interval/Coding strand**<br>**c) Pfam/BLAST analysis (e-values) of predicted sequence**<br>**d) Fig. in main text**	**Predicted amino acid sequence from AnaBlast**
a) Chromosome II: 4448915-445307<br>b) 4452616-4452379/Reverse strand<br>c) Chimeric coding sequence. 4452616-4452443. BLAST: fragment of amino-acid permeases. *S. pombe* ref|NP_596849.1| (2e-^{20})<br>+ 4452444-4452379. BLAST: small bacterial transposase fragment. *Desulfobacter postgatei* ref|WP_004074224.1| 73% identity<br>d) Fig. 7C	N G V D A S P F V L T I K D V N I K K L P S I L S A I V L I F V I S S A N A R L Y A G S S A I H S L G C N G F<br>+<br>M K Q R G E T Y L Y I Y N C F L S C F L H I Stop
a) Chromosome II: 36586-38588<br>b) 36890-37298/Forward strand<br>c) BLAST: maturase protein 2 domain- fission yeast mitochondrion. Sequence ID: pir|| S78197. (5e^{-56})<br>d) Fig. 8A	Met F D F G I V R K I K D K N A I L F I I A N S K G I E R V I T L I N N K F R T T S K Y N Q I I N N I F A Q P R F K E F S K T I N L S L N T F Q L S V L N N H W L A G F S D A D A S F Q I K I L N R D X K I E V R L N Y Q I D Q K K E Y L L S L I K D N L N K I Q L I L E S G K Stop
a) Chromosome II: 4090826-4092326<br>b) 4091456-4091554/Forward strand<br>c) BLAST: transposase IS66 family protein (*Bacillus mycoides*) ref: gb|KFN12866.1| (58% identity).<br>d) Fig. 8B	M K R M N L L K K L Q R L M F Y K K S F H G S G L C E I N F R R
a) Chromosome II: 2433482-2435634<br>b) 22433875- 22433935/Forward strand<br>c) Pfam: Cytochrome c oxidase subunit III (1.49e^{-05})<br>BLAST: cytochrome c oxidase subunit III (*Hylarana sp*. FRIM 1401)<br>(1e^{-07})<br>d) Fig. 8C	T Y W H F Y D V V W L F L Y L T I Y I I
a) Chromosome II: 2958430-2959079<br>b) 2958676-2958843/Reverse strand<br>c) Pfam: Alpha amylase, catalytic domain (0.0016)<br>BLAST: trehalose synthase, partial (*Streptomyces chartreusis*)<br>ref|WP_010033287.1| (1e^{-17})<br>d) Fig. 8D	A L I K E V V E I V N Y L T T Q G I D S I R L D T V Y H L Y E L S R V N F K K Y P I T K K D K T S I H R F F Q K
